# Nowe Zalecenia Dotyczące Diagnostyki i Leczenia Eozynofilowego Zapalenia Przełyku u dzieci i Dorosłych

**DOI:** 10.34763/devperiodmed.20182204.379384

**Published:** 2019-01-14

**Authors:** Barbara Iwańczak, Franciszek Iwańczak

**Affiliations:** 1 II Katedra i Klinika Pediatrii, Gastroenterologii i Żywienia, Uniwersytet Medyczny im. Piastów Śląskich we Wrocławiu, Wrocław Polska

**Keywords:** eozynofilowe zapalenie przełyku, epidemiologia, diagnostyka, leczenie, dzieci, dorośli, eosinophilic esophagitis, epidemiology, diagnosis, treatment, children, adults

## Abstract

Eozynofilowe zapalenie przełyku (EoE) jest obok choroby refluksowej najczęściej rozpoznawaną przewlekłą chorobą zapalną przełyku, występującą zarówno u dzieci jak i u pacjentów dorosłych. Objawy kliniczne EoE zależą od wieku pacjenta i czasu trwania choroby. U niemowląt i młodszych dzieci występują zaburzenia karmienia, upośledzenie rozwoju fizycznego, wymioty, bóle brzucha. U dzieci starszych i dorosłych dominują zaburzenia połykania pokarmów stałych, zatrzymanie kęsa pokarmowego w przełyku, bóle w klatce piersiowej. W obrazie endoskopowym przełyku stwierdza się zmiany zapalne błony śluzowej, bruzdy, pierścienie i błony oraz następowe zwężenie światła przełyku. W badaniu histologicznym stwierdza się zapalenie przełyku z naciekiem komórek kwasochłonnych. W 2017 roku międzynarodowa robocza grupa ekspertów pod kierunkiem A.J. Lucendo i auspicjami towarzystw naukowych: UEG – United European Gastroenterology, ESPGHAN – European Society of Pediatric Gastroenterology, Hepatology and Nutrition, EAACI – European Academy of Allergy and Clinical Immunology, EUREOS – Eupropean Society of Eosinophilic Oesophagitis, opracowała zalecenia diagnostyczne i lecznicze EoE, stosując metodę GRADE (Grading of Recommendations Assessment, Development and Evaluation). Międzynarodowa grupa ekspertów składała się z gastroenterologów, alergologów, pediatrów, laryngologów, patologów i epidemiologów. Szeroki udział specjalistów z różnych dziedzin medycyny pozwolił w opracowanych zaleceniach uwzględnić różne aspekty tej choroby.

## Abstract (en)

Besides gastro-esophageal reflux, eosinophilic esophagitis (EoE), is the most frequently diagnosed chronic inflammatory disease both in children and adults. Clinical symptoms depend on the age of the patient and the time of the disease. In infants and younger children feeding disturbances, physical development retardation, vomiting and abdominal pain are most frequent. Difficulty in solid food swallowing, food impaction in the esophagus and chest pain are the most frequent symptoms in older children and adults. In the endoscopic study of the esophagus, esophagitis, rings and esophageal stricture may be visible. Traits of chronic inflammation and eosinophilic infiltration are present in histologic examination. In the year 2017, an international experts’ working group, headed by A.J. Lucendo, under the auspices of UEG - United European Gastroenterology, ESPGHAN - European Society of Pediatric Gastroenterology, Hepatology and Nutrition, EAACI – European Academy of Allergy and Clinical Immunology, EUREOS - European Society of Eosinophilic Oesophagitis, elaborated diagnostic and therapeutic recommendations for eosinophilic esophagitis using the GRADE method (Grading of Recommendations Assessment, Development and Evaluation). The international experts’ group included gastroenterologists, aerologists, pediatricians, laryngologists, pathologists and epidemiologists. The wide spectrum of specialists in various fields made it possible to consider various aspects of the disease in their recommendations.

## Wprowadzenie

Eozynofilowe zapalenie przełyku (EoE) jest przewlekłą chorobą zapalną charakteryzującą się dysfunkcją przełyku i naciekiem zapalnym błony śluzowej przełyku z dominacją granulocytów kwasochłonnych. Eozynofilowe zapalenie przełyku zostało po raz pierwszy opisane w 1978 roku przez Landresa i wsp. [1], a następnie zdefiniowane jako oddzielny zespół chorobowy w latach 1993 i 1994 [2, 3]. Od tego czasu obserwuje się znaczny wzrost zainteresowań tą chorobą, jej objawami klinicznymi, patomechanizmem oraz leczeniem. Znacznie wzrosła liczba rozpoznań tej choroby zarówno u dzieci jak i pacjentów dorosłych, częściej u chłopców i młodych mężczyzn, z wywiadem obciążonym chorobami alergicznymi [4, 5, 6]. W Polsce, w 2007 roku ukazał się pierwszy opis eozynofilowego zapalenia przełyku i dotyczył 18-letniego pacjenta, u którego po spożyciu stałego posiłku wystąpiło uczucie zatrzymania pokarmu w przełyku, trudności w połykaniu pokarmów stałych, bóle w klatce piersiowej. Badanie rtg kontrastowe przełyku wykazało na wysokości łuku aorty zatrzymanie kontrastu. Badanie ezofagogastrofiberoskopowe oraz badanie histologiczne wycinka błony śluzowej przełyku potwierdziły rozpoznanie eozynofilowego zapalenia przełyku [7]. W 2011 roku przeprowadzono w Polsce wieloośrodkowe badanie dotyczące częstości występowania choroby i jej manifestacji klinicznej, endoskopowej oraz sezonowości rozpoznawania EoE u dzieci do 18 roku życia [5]. Analiza 84 dzieci z rozpoznaniem eozynofilowego zapalenia przełyku wykazała częstsze występowanie u chłopców w porównaniu z dziewczynkami (64/20). Eozynofilowe zapalenie przełyku było częściej rozpoznawane u dzieci po 10 roku życia, a najmłodszy pacjent miał 4 miesiące. EoE było częściej rozpoznawane w okresie wiosenno-letnim (45,2%/28,5%), a także u dzieci z chorobami alergicznymi w wywiadzie. Częstość rozpoznawania EoE wynosiła 1/424 badania endoskopowe górnego odcinka przewodu pokarmowego [5]. Noel i wsp. [8] analizując częstość występowania choroby w populacji pediatrycznej wykazali, że w 2003 roku częstość ta wynosiła 1,28 na 10 000 mieszkańców. Badania epidemiologiczne prowadzone w ostatnich dekadach zwracają uwagę na wzrost zachorowań zarówno u dzieci jak i dorosłych [4]. Eozynofilowe zapalenie przełyku jest więc jedną z najczęstszych chorób przełyku i wiodącą przyczyną dysfagii i zatrzymania pokarmu stałego w przełyku. W patogenezie ważną rolę odgrywają czynniki genetyczne, środowiskowe oraz immunologiczne. Wykazano częstsze występowanie EoE u osób płci męskiej oraz występowanie rodzinne [9, 10]. U chorych wykazano polimorfizm pojedynczego nukleotydu w genie na choromosomie 7, kodującym eotaksynę 3, która odgrywa istotną rolę w patogenezie choroby. Dużą rolę przypisuje się czynnikom środowiskowym w tym alergenom pokarmowym i wziewnym, czynnikom infekcyjnym (*Herpes simplex virus, mycoplasma, H. pylori*), stosowaniu antybiotyków, inhibitorów pompy protonowej i in. W immunopatogenezie EoE alergeny pokarmowe i wziewne powodują odpowiedź limfocytów Th2, cytokin, pobudzenie mastocytów i aktywacje czynników mających potencjalny udział w *remodelingu* przełyku [11].

W kilku wcześniejszych zaleceniach opracowanych przez ekspertów towarzystw naukowych zostały przedstawione kryteria diagnostyczne i zalecenia lecznicze tej choroby [9, 10, 11, 12, 13, 14, 15]. W 2011 roku ustalono, że do rozpoznania EoE wymagana jest obecność 15 i więcej eozynofilii w błonie śluzowej przełyku przy dużym powiększeniu [9]. Zmieniło się także podejście dotyczące różnicowania EoE z chorobą refluksową przełyku i leczenia inhibitorami pompy protonowej [16, 17, 18, 19].

## Nowe zalecenia diagnostyczne i lecznicze

Najnowsze rekomendacje dotyczące diagnostyki i leczenia EoE u dzieci i dorosłych zostały opublikowane w 2017 roku [15]. Wymienione zalecenia zostały opracowane przez międzynarodową grupę roboczą złożoną z ekspertów pod kierunkiem Lucendo i pod auspicjami towarzystw naukowych. W skład tej grupy weszli specjaliści z dziedziny gastroenterologii, pediatrii, laryngologii, patologii i epidemiologii. ZZaalleecceenniiaa ddiiaaggnostyczne i lecznicze EoE zostały opracowane metodą GRADE (Grading of Recommendations Assessment, Development and Evaluation).

## Definicja

Eozynofilowe zapalenie przełyku jest przewlekłą chorobą związaną z odpowiedzią immunologiczną przełyku, której towarzyszą zmiany histologiczne ściany przełyku z miejscowym naciekiem zapalnym, z dominacją eozynofilii oraz różnorodne, zależne od wieku i czasu trwania zapalenia objawy kliniczne spowodowane dysfunkcją przełyku. W diagnostyce tej choroby należy wykluczyć inne przyczyny eozynofilii miejscowej i systemowej, a objawy kliniczne i zmiany histopatologiczne nie powinny być interpretowane oddzielnie. Wycofane zostały z poprzedniej definicji określenia, że EoE jest chorobą przełyku „zależną od antygenu” oraz pojęcie „eozynofilii przełyku odpowiadającej na leczenie inhibitorami pompy protonowej” (PPI – REE: Proton Pump Inhibitor –Responsive Esophageal Eosinophilia), stosowanego od 2011 roku jako kryterium diagnostyczne, gdyż jedynie pacjenci nieodpowiadający na terapię PPI albo alternatywnie z prawidłową pH-metrią przełyku mogli mieć rozpoznane EoE. Według tych założeń tylko GERD (Gastroesophageal Reflux Disease)i choroby zależne od kwasu solnego odpowiadają na leczenie PPI. Obecnie uważa się, że remisja kliniczna i histologiczna na terapii PPI jest raczej częścią przebiegu EoE niż oddzielną jednostką chorobową, a pacjenci odpowiadający i nieodpowiadający na terapię PPI mają nakładające się cechy fenotypowe i genetyczne [9, 15].

## Epidemiologia

Eozynofilowe zapalenie przełyku jest rozpoznawane w każdym wieku. U dzieci częstość rozpoznań wzrasta wraz z wiekiem, u pacjentów dorosłych szczyt zachorowań występuje pomiędzy 30-50 rokiem życia. W zależności od szerokości geograficznej zachorowalność na EoE wynosi od 3 do 13 nowych zachorowań na 100 000 mieszkańców/rok w Europie, USA i Kanadzie [20]. Natomiast chorobowość waha się pomiędzy 40-56 przypadków na 100 000 mieszkańców w Europie i USA. U dzieci częstość występowania choroby wynosi od 0,7 do 10/100 000/rok, a chorobowość od 0,2 do 43/100 000. W ostatnich dekadach obserwuje się wzrost zachorowań na eozynofilowe zapalenie przełyku zarówno u dzieci jak i dorosłych [4, 20]. Eozynofilowe zapalenie przełyku częściej jest rozpoznawane u mężczyzn rasy białej i u pacjentów chorujących na choroby alergiczne [5, 6].

## Objawy kliniczne

Obraz kliniczny eozynofilowego zapalenia przełyku zależy od wieku pacjenta i fenotypu choroby. U niemowląt i młodszych dzieci najczęstszymi objawami są: niepokój, trudności w karmieniu i odmowa przyjmowania pokarmu, ulewanie, wymioty oraz ból w nadbrzuszu, prowadzące do zahamowania rozwoju dziecka. U pacjentów starszych podobnie jak u dorosłych występują zaburzenia połykania pokarmów stałych z epizodami zaklinowania kęsa pokarmu (food impaction) w przełyku, zgaga, ból w klatce piersiowej. Pacjenci często starają się pokonać istniejące zaburzenia w połykaniu pokarmów stałych popijaniem posiłków wodą, unikają pokarmów stałych, co przyczynia się do zmniejszenia dolegliwości a także do opóźnienia wykonania badania endoskopowego i rozpoznania choroby. U osób dorosłych wśród objawów choroby dysfagia występuje w 70-80%, zatrzymanie pokarmu w 33-54% [15]. W badaniu endoskopowym przełyku możemy obserwować wysięk z białymi nalotami, linijne bruzdy, okrężne pierścienie (trachealizacja), obrzęk, bladość błony śluzowej przełyku oraz rzadziej występujące u dzieci zwężenie przełyku. Ponadto może występować przełyk w postaci bibuły karbowanej, krucha błona śluzowa wrażliwa na dotyk w czasie manipulacji endoskopem. Hirano i wsp. [21] opracowali dla pacjentów dorosłych skalę zmian endoskopowych przełyku, w której uwzględnione są następujące zmieny: wydzielina, naloty (exudates, plagues), bruzdy (furrowes), pierścienie (rings) oraz obrzęk i bladość (edema, pallor) błony śluzowej. Wymieniona skala jest także zalecana do stosowana u dzieci. U pacjentów z podejrzeniem EoE wskazane jest pobranie w czasie badania endoskopowego do oceny histologicznej co najmniej 6 wycinków błony śluzowej z różnych części przełyku zarówno z proksymalnej jak i dystalnej, zwłaszcza w obrębie zmian endoskopowych. Zgodnie z przyjętym konsensusem do rozpoznania EoE niezbędna jest obecność co najmniej 15 eozynofilii w polu widzenia przy dużym powiększeniu (hpt) (ok. 0,3 mm^2^), tzn. powiększeniu mikroskopowym 400-krotnym. Ponadto w badaniu histologicznym bioptatu błony śluzowej przełyku mogą występować mikroropnie złożone z eozynofilii (skupiska co najmniej 4 eozynofilii), przerost i/lub włóknienie warstwy podstawnej, poszerzenie przestrzeni międzykomórkowej, wydłużenie warstwy brodawkowej. Należy zaznaczyć, że objawy kliniczne nie korelują dobrze z histologiczną aktywnością EoE. Dlatego niezbędne jest badanie histopatologiczne do monitorowania choroby.

## Różnicowanie

Eozynofilowe zapalenie przełyku należy różnicować z innymi chorobami przebiegającymi z eozynofilią przełyku jak: choroba refluksowa przełyku, choroby infekcyjne przełyku, eozynofilowe zapalenie żołądka i jelita cienkiego, choroba trzewna, achalazja, choroby tkanki łącznej, HES (Hypereosinophilic Syndrome), nadwrażliwość na leki i inne. W chorobie reffluksowej przełyku możemy obserwować niewielką eozynofilię przełyku, nie przekraczającą 5/eozynofilii/hpt. Należy podkreślić, że EoE i GERD stanowią odrębne jednostki chorobowe, mimo podobnych objawów, które mogą występować niezależnie od siebie, względnie mogą się na siebie nakładać.

## Historia naturalna EoE

Nieleczone eozynofilowe zapalenie przełyku najczęściej prowadzi do występowania przewlekłych objawów chorobowych związanych z dysfunkcją przełyku spowodowaną zapaleniem, które może powodować przebudowę ściany przełyku (*remodeling*), zwłóknienie, zwężenie i zaburzenie połykania. Leczenie przeciwzapalne eozynofilowego zapalenia przełyku może ograniczyć progresję choroby. Eozynofilowe zapalenie przełyku ma wpływ na jakość życia pacjentów poprzez negatywne oddziaływania psychologiczne na aktywność fizyczną i społeczną. Brak jest dowodów, że EoE jest stanem chorobowym, który może prowadzić do rozwoju nowotworu przełyku.

## Leczenie

W leczeniu eozynofilowego zapalenia przełyku zarówno u dzieci jak i pacjentów dorosłych stosujemy leczenie dietetyczne, farmakologiczne oraz w przypadku zwężenia przełyku endoskopowe rozszerzanie przełyku. Wybór terapii powinien być indywidualnie przedyskutowany z pacjentem lub rodzicami dziecka i może być zmieniony, zależnie od wyniku leczenia, wraz z upływem czasu. Skuteczność każdej z terapii tzn. dietetycznej czy farmakologicznej powinna być oceniana endoskopowo po 6-12 tygodniach leczenia. Celem terapii jest ustąpienie objawów klinicznych oraz zmian zapalnych błony śluzowej przełyku. Leczenie powinno być monitorowane poprzez ocenę wycofywania się objawów klinicznych i kontrolne badanie endoskopowe z oceną histopatologiczną. Jednak wieloletnia obserwacja dzieci wykazała brak korelacji pomiędzy ustępowaniem objawów klinicznych pod wpływem leczenia, a zmianami zapalnymi i włóknieniem przełyku. Dieta eliminacyjna, inhibitory pompy protonowej, kortykosteroidy działające miejscowo powinny być proponowane jako leczenie przeciwzapalne pierwszego rzutu.

### Leczenie dietetyczne

Empiryczna dieta eliminująca 6 alergenów pokarmowych (mleko, gluten, jajka, soja, orzechy, ryby) indukuje remisję histologiczną u około ¾ chorych dzieci i dorosłych. U pacjentów dorosłych eliminacja 4 alergenów pokarmowych indukowała remisję u około 50% chorych, natomiast eliminacja 2 alergenów (mleko i gluten) u 40% pacjentów. Bardzo często odpowiednia eliminacja alergenów pokarmowych pozwala zatem na utrzymanie remisji wolnej od leczenia farmakologicznego, co jest dużą zaletą. Należy zaznaczyć, że przydatność testów alergicznych w identyfikacji alergenów jest dość niska. Dobre efekty obserwowano także po zastosowaniu diety elementarnej [21]. Dieta elementarna (preparaty aminokwasów) indukuje remisję histologiczną u około 90% dzieci i dorosłych z eozynofilowym zapaleniem przełyku. Dieta elementarna ma jednak ograniczone zastosowanie ponieważ posiada niskie walory smakowe i z tego powodu często bywa konieczność jej stosowania poprzez sondę nosowo-żołądkową, co znacznie obniża jakość życia pacjentów. Ponadto dieta elementarna jest droga i stanowi wysoki koszt leczenia, gdyż mieszanki elementarne nie są refundowane przez NFZ. Stosowanie diety elementarnej powinno być ograniczone i rozważane tylko po niepowodzeniu prawidłowo prowadzonego leczenia dietą eliminacyjną i/lub leczenia farmakologicznego. Należy podkreślić, że większą skuteczność leczenia dietetycznego obserwuje się u pacjentów z wywiadem atopowym.

### Kortykosteroidy

Zarówno u dzieci jak i u pacjentów dorosłych skuteczną indukcję histologiczną EoE uzyskuje się kortykosteroidami podawanymi miejscowo do przełyku. Terapia jest najbardziej skuteczna gdy lek jest stosowany w postaci połykanego lepkiego syropu, wówczas kontakt leku z dystalną częścią przełyku jest dłuższy. Zalecany jest *flutykazon*, który w indukcji remisji u dzieci stosuje się w dawce 880-1760 μg/dobę, a u pacjentów dorosłych w dawce 1760 μg/dobę. Dawki podtrzymujące wynoszą: u dzieci 440-880 μg/dobę, u dorosłych 880-1760 μg/dobę. Kortykosteroidy stosowane miejscowo są bardziej bezpieczne i mają mniej działań niepożądanych w porównaniu ze stosowanymi w szczególnie ciężkich przypadkach glikokortykosterydami systemowo. Z tych glikokortykosteroidów - *budesonid* w indukcji remisji u dzieci jest stosowany w dawce 1-2 mg/dobę, natomiast 2-4 mg/dobę u dorosłych. W podtrzymaniu remisji dawka wynosi:1mg/dobę u dzieci i 2mg/dobę u pacjentów dorosłych. Jednak glikokortykosteroidy stosowane ogólnie nie są rekomendowane w EoE [15].

### Inhibitory pompy protonowej

Liczne badania wykazały, że terapia inhibitorami pompy protonowej (PPI-Proton Pump Inhibitor) jest skuteczna w indukcji remisji EoE zarówno u dzieci jak i pacjentów dorosłych. U pacjentów obserwowano zarówno ustąpienie objawów klinicznych (ponad 60%) jak i eozynofilii (ponad 50%). Rekomendowane dawki *omeprazolu*: u dzieci 1-2 mg/kg/dobę podzielone w dwóch dawkach; dorośli 20-40 mg/2 x dobę. Wykazano większą skuteczność PPI, u pacjentów z nieprawidłowym zapisem pH-metrii oraz gdy PPI był stosowany 2 x dobę w porównaniu ze stosowaniem 1 x dobę. U pacjentów, którzy odpowiedzieli na leczenie PPI długotrwała terapia podtrzymująca PPI była skuteczna w podtrzymaniu remisji [15]. Jednak u większości pacjentów po przerwaniu leczenia obserwowano nawrót objawów klinicznych i/lub histopatologicznych [17].

Wymienione leczenie dietą eliminacyjną, inhibitorami pompy protonowej czy miejscowo kortykosteroidami jest proponowane jako leczenie przeciwzapalne pierwszego rzutu. U pacjentów z dysfagią, utknięciem kęsa pokarmowego w przełyku, nieodpowiadających na leczenie przeciwzapalne powinno być wykonane endoskopowe rozszerzanie przełyku. Endoskopowe rozszerzanie przełyku zmniejsza zaburzenia połykania u ponad ¾ dorosłych pacjentów ze zwężonym światłem przełyku, natomiast pozostaje bez wpływu na zapalenie przełyku. W wybranych ciężkich przypadkach EoE nie reagujących na wymienione metody leczenia przeciwzapalnego mogą być przydatne zarówno w indukcji jak i utrzymaniu długotrwałej remisji: azatiopryna i 6-merkaptopuryna. Stosowanie przeciwciał przeciwko interleukinie-5, przeciwciał przeciwko Il-13 nie miało wpływu na objawy EoE i w niewielkim stopniu zmniejszało eozynofilię przełyku. Stosowane przeciwciała przeciwko IgE (*omalizumab*), przeciwko TNF-alfa (infliximab), jak również kromoglikan sodu i leki przeciwhistaminowe nie miały wpływu na objawy i eozynofilię przełyku, podobnie jak brak jest wystarczających dowodów aby rekomendować antagonistę receptora leukotrienowego (*montelukast*).

Na rycinie 1 przedstawiono algorytm leczenia w eozynofilowym zapaleniu przełyku wg Lucendo i wsp. [15].

**Ryc. 1:**
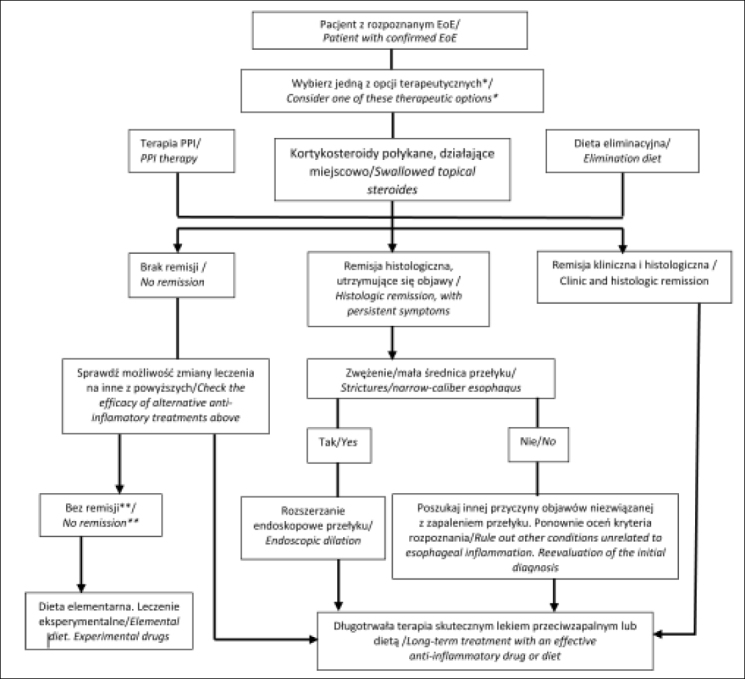
Algorytm terapeutyczny pacjentów z rozpoznanym eozynofilowym zapaleniem przełyku [wg 15]. Fig. 1. Therapeutic algorithm proposed for eosinophilic esophagitis in clinical practice [acc.15]. *U pacjentów z utrzymującymi się objawami po terapii przeciwzapalnej należy rozważyć rozszerzanie przełyku/*In patients with persistent symptoms under anti-inflammatory therapy, endoscopic dilation should be considered* **Skieruj pacjenta do ośrodka referencyjnego/*Refer the patient to an EoE center*
